# Novel Epitopes of the Influenza Virus N1 Neuraminidase Targeted by Human Monoclonal Antibodies

**DOI:** 10.1128/jvi.00332-22

**Published:** 2022-04-21

**Authors:** Ericka Kirkpatrick Roubidoux, Kaori Sano, Meagan McMahon, Juan Manuel Carreño, Christina Capuano, Kaijun Jiang, Viviana Simon, Harm van Bakel, Patrick Wilson, Florian Krammer

**Affiliations:** a Department of Microbiology, Icahn School of Medicine at Mount Sinai, New York, New York, USA; b Graduate School of Biomedical Sciences, Icahn School of Medicine at Mount Sinai, New York, New York, USA; c Global Health Emerging Pathogens Institute, Icahn School of Medicine at Mount Sinai, New York, New York, USA; d Division of Infectious Diseases, Department of Medicine, Icahn School of Medicine at Mount Sinai, New York, New York, USA; e Department of Genetics and Genomic Sciences, Icahn School of Medicine at Mount Sinai, New York, New York, USA; f Icahn Institute for Data Science and Genomic Technology, Icahn School of Medicine at Mount Sinai, New York, New York, USA; g Department of Medicine, Section of Rheumatology, the Knapp Center for Lupus and Immunology, University of Chicago, Chicago, Illinois, USA; University of California, Irvine

**Keywords:** N1, influenza, mAbs, neuraminidase

## Abstract

Influenza virus neuraminidase (NA)-targeting antibodies are an independent correlate of protection against influenza. Antibodies against the NA act by blocking enzymatic activity, preventing virus release and transmission. As we advance the development of improved influenza virus vaccines that incorporate standard amounts of NA antigen, it is important to identify the antigenic targets of human monoclonal antibodies (mAbs). Here, we describe escape mutants generated by serial passage of A/Netherlands/602/2009 (H1N1)pdm09 in the presence of human anti-N1 mAbs. We observed escape mutations on the head domain of the N1 protein around the enzymatic site (S364N, N369T, and R430Q) and also detected escape mutations located on the sides and bottom of the NA (N88D, N270D, and Q313K/R). This work increases our understanding of how human antibody responses target the N1 protein.

**IMPORTANCE** As improved influenza virus vaccines are being developed, the influenza virus neuraminidase (NA) is becoming an important new target for immune responses. By identifying novel epitopes of anti-NA antibodies, we can improve vaccine design. Additionally, characterizing escape mutations in these epitopes aids in identifying NA antigenic drift in circulating viruses.

## INTRODUCTION

Influenza viruses cause seasonal epidemics and, occasionally, global pandemics that lead to significant morbidity and mortality worldwide (1, 2). They are a member of the family *Orthomyxoviridae* and contain a segmented, negative-sense RNA genome. Two of the genomic segments encode the glycoproteins present on the viral surface, the hemagglutinin (HA) and the neuraminidase (NA) (3, 4). The HA of influenza viruses, which is responsible for receptor binding and viral entry, has been largely credited as the immunodominant target of the antibody response after vaccination and natural infection (3–5). The NA acts as a sialidase, removing terminal sialic acids and allowing viral egress and spread. It has recently become appreciated as an additional important target of anti-influenza virus immunity (6–9). To function properly, the NA must be present on the viral surface as a homotetramer (10–12).

Seasonal influenza virus vaccines are the first line of defense against infection (13). Typically, these vaccines are standardized based on the HA content but have varying NA content with unknown structural integrity (14, 15). In addition, seasonal vaccines can have varying effectiveness from 20% to 60% in a given year (16). Low vaccine effectiveness can be largely attributed to the antigenic variability of the HA vaccine component compared to circulating strains (17–20). It may be possible to improve seasonal vaccine effectiveness by including a standard amount of a second viral antigen, the NA (7, 8). During natural infection, antibodies targeting both the HA and the NA are produced; however, NA antibodies are rarely detected after vaccination (14). NA-specific antibodies have been demonstrated to prevent severe infections, restrict transmission, and protect from lethal challenge in the mouse model (12, 21–26). These antibodies often function as NA inhibitors by blocking the NA enzymatic site and preventing viral spread (14, 21).

Residues critical for NA-inhibiting antibodies were first characterized using murine antibodies (27–29). The monoclonal antibody (mAb) CD6 was found to span the dimer interface, while other mAbs were found to bind to only a single monomer. Additional work has been ongoing to identify targets of human mAbs (14, 30–32). A majority of these residues can be attributed to the discovery of broadly reactive NA mAbs that target the enzymatic site (32). Interestingly, few residues have been identified as targets of both human and murine mAbs (these include residues 248, 249, 270, 273, 309, 369, 451, and 456 when numbering from methionine). This emphasizes the importance of mapping epitopes of human mAbs onto the N1 protein. The targets of several previously published mAbs have yet to be defined, leaving a gap in our understanding. Here, we use a panel of these uncharacterized mAbs to determine additional N1 residues targeted by human anti-N1 mAbs. The mAbs used in this study were isolated from individuals who were naturally infected and had various levels of cross-reactivity and neuraminidase inhibition (NAI) activity (14).

## RESULTS

### Generation of N1 mAb escape mutant viruses.

For epitope analysis, we chose a panel of N1-specific mAbs from a recently published study (14). A detailed description of the mAbs, including information about their complementarity-determining regions (CDRs), was reported previously (14). Our panel consisted of 8 mAbs: EM-2E01, 1000-1D05, 1000-3B04, 1000-3B06, 1000-3C05, 294-16-009-A-1C02, 294-16-009-A-1D05, and 300-16-005-G-2A04. We also included a negative IgG control antibody, KL-1C12, which targets the Ebola virus glycoprotein, and two control “irrelevant IgG control viruses” (A and B) were derived from passaging virus with this antibody present (33). Virus passaged in the same cells in the presence of an irrelevant mAb serves as stringent control since it will also pick up relevant cell culture-adaptive mutations or changes triggered by the presence of nonspecific IgG. Irrelevant IgG control virus A shared many HA mutations with the escape mutant viruses (EMVs); however, it also contained a mutation in the NA (D454G). Irrelevant IgG control virus B contained a unique HA stalk mutation (E391G) but contained no NA mutations, making it more desirable for *in vitro* experiments. Each mAb’s neuraminidase inhibition (NAI) activity, measured using an enzyme-linked lectin assay (ELLA), and neutralization activity, measured by a plaque reduction neutralization assay (PRNA), were first determined against the wild-type A/Netherlands/602/2009 (H1N1)pdm09 strain. All mAbs, aside from 1000-3C05 and 294-16-009-A-1D05, had NAI activity (Table 1). The mAb 300-16-005-G-2A04 did not have neutralization activity, and mAbs 1000-3C05, 294-16-009-A-1C02, and 294-16-009-A-1D05 had low neutralization activity (Table 1).

**TABLE 1:** NAI and neutralization activities of mAbs against wild-type A/Netherlands/602/2009 (H1N1)pdm09 virus^*a*^

mAb	NAI IC_50_ (μg/mL) (95% confidence interval)	PRNA IC_50_ (μg/mL) (95% confidence interval)
EM-2E01	0.027 (0.022–0.031)	0.034 (0.031–0.038)
1000-1D05	0.64 (0.45–0.58)	2.66 (1.42–5.03)
1000-3B04	0.95 (0.81–1.13)	3.66 (3.58–3.74)
1000-3B06	0.27 (0.22–0.34)	0.15 (0.14–0.16)
1000-3C05	>30	23.40 (14.64–39.91)
294-16-009-A-1C02	9.2 (7.92–10.74)	14.43 (11.57–17.62)
294-16-009-A-1D05	>30	35.52 (27.27–46.87)
300-16-005-G-2A04	0.097 (0.076–0.123)	>100
1C12	NA	NA

a IC_50_ values (with 95% confidence intervals) are listed in micrograms per milliliter. NA, not applicable.

**FIG 1:**
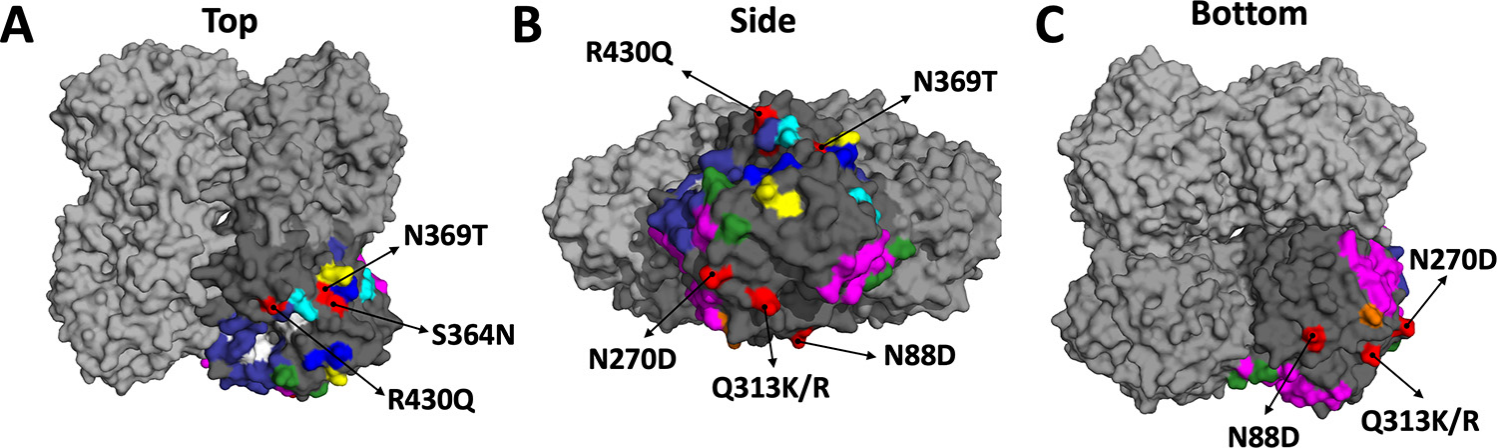
Escape mutations mapped onto a three-dimensional structure of the NA. The NA of A/California/04/2009 (PDB accession number 3NSS [45]) is depicted as a tetramer with 3 monomers in light gray and one in darker gray. The darker gray subunit has residues identified in previous publications and the NA active site (in white). Murine epitopes are illustrated in blue (27), magenta (28), and yellow (29). Human epitopes are indicated in orange (14), cyan (31), green (30), and indigo (32). Mutations identified in the EMVs used for this study are highlighted in red and identified using arrows. Views from the top (A), side (B), and bottom (C) of the NA are depicted.

EMVs were produced by passaging the wild-type A/Netherlands/602/2009 (H1N1)pdm09 virus with each antibody in Madin-Darby canine kidney (MDCK) cells. We began with multiplicities of infection (MOIs) of 0.01 and 0.25 times the 50% inhibitory concentration (IC_50_) of each mAb. For NAI-active mAbs, the NAI IC_50_ was used, while the PRNA IC_50_ was used for NAI-inactive mAbs. At each subsequent passage, we doubled the amount of mAb present in the medium. EMVs were detected after 4 to 10 passages (2× IC_50_ to 128× IC_50_) (Table 2). mAbs EM-2E01, 1000-3B04, 1000-3C05, 294-16-009-A-1C02, and 300-16-005-G-2A04 generated 7 distinct EMVs. The NA mutations identified were N88D (1000-3C05), N270D (1000-3B04), Q313K/R (294-16-009-A-1C02), S364N (EM-2E01), S364N/N369T (EM-2E01), and R430Q (300-16-005-G-2A04). Additionally, one of the irrelevant IgG control viruses contained NA mutation D454G (Table 2). We did not detect any NA mutations in virus isolated from the cell culture supernatant supplemented with the mAbs 1000-1D05, 1000-3B06, and 294-16-009-A-1D05 after 10 passages. The detected mutations are distributed in different regions of the NA protein (Fig. 1). S364N, N369T, and R430Q are located on the top of the tetramer (Fig. 1A). N270D and Q313K/R are located on the side of the tetramer (Fig. 1B and C). N88D is located on the bottom of the tetramer, near the head/stalk interface (Fig. 1C), and R430Q is the closest to the NA enzymatic site. Mutations at N270 and N369 have also been identified using human mAbs in other studies (14, 30). The mutations N88D, Q313K/R, S364N, and R430Q have not been previously identified using human mAbs. Each EMV and one of the irrelevant IgG control viruses shared several HA mutations (Table 2). Mutations in other genomic segments were also found (Table 2).

**TABLE 2:** Mutations identified in passaged viruses^*a*^

EMV	mAb	No. of passages to escape	NA mutation(s)	PB2 mutation(s)	PB1 mutation(s)	PA mutation(s)	HA mutation(s)	NP mutation(s)	M mutation(s)	NS1 mutation(s)
N88D	1000-3C05	8	N88D	ND	ND	**V100L**	**R62K**, **D239G**, **R240Q**	E372D	E204D	ND
N270D	1000-3B04	9	N270D	ND	ND	**V100L**	**R62K**, K136N, **D239G**, **R240Q**	ND	ND	ND
Q313K	294-16-009-A-1C02	4	Q313K	ND	ND	ND	**R62K**, K136N, **D239G**, **R240Q**	ND	ND	ND
Q313R	294-16-009-A-1C02	4	Q313R	ND	ND	ND	**R62K**, **D239G**, **R240Q**	S50N	ND	ND
S364N	EM-2E01	6	S364N	ND	ND	**V100L**	**R62K**, **D239G**, **R240Q**	ND	ND	ND
S364N/N369T	EM-2E01	6	S364N/N369T	ND	ND	**V100L**	**R62K**, **D239G**, **R240Q**	ND	ND	ND
R430Q	300-16-005-G-2A04	10	R430Q	ND	ND	**V100L**	K226M	S50N	ND	ND
A/New York/PV01575/2018	NA	NA	V13I, V34I, L40I, N44S, G77R, V81A, V108I, I188T, N200S, N222D, V241I, N248D, V264I, N270K, I314M, V321I, N369K, N386K, D416N, K432E, N449D	R54K, M66I, A184V, D195N, R293K, R299K, V344M, I354L, S453T, V731I	G154D, I397M, I435T	G66D, V100I, K142E, N321K, I330V, G349E, R362K, L581M	A13T, S91R, S101N, D114N, D173N, S179N, K180Q, S181T, S200P, S202T, S220T, I233T, **R240Q**, E252D, A273T, N277D, K300E, I312V, **E391K**, S468N, E516K, V537A	A22T, E53D, V100I, M105T, N247S, I373T, S498N	V80I, M192V, Q208K, K230R	D2E, E55K, L90I, I123V, E125D, K131E, N205S
Irrelevant IgG control virus A^*b*^	1C12	6	D454G	ND	ND	V407I	R62K, K163N, D239G, R240Q	ND	ND	G179R, I198L
Irrelevant IgG control virus B	1C12	10	ND	ND	ND	V100L	E391G	ND	ND	ND

a Each mAb and the mutations in its corresponding EMV are listed. ND, none detected; NA, not applicable. Numbering starts from the methionine of each protein. Mutated residues shared between EMVs and A/New York/PV01575/2018 are underlined. Mutated residues in boldface type are shared with irrelevant IgG control virus A or B.

b Also reported in reference 34.

### Escape mutant viruses are resistant to binding, NAI, and neutralization activities of mAbs.

**FIG 2:**
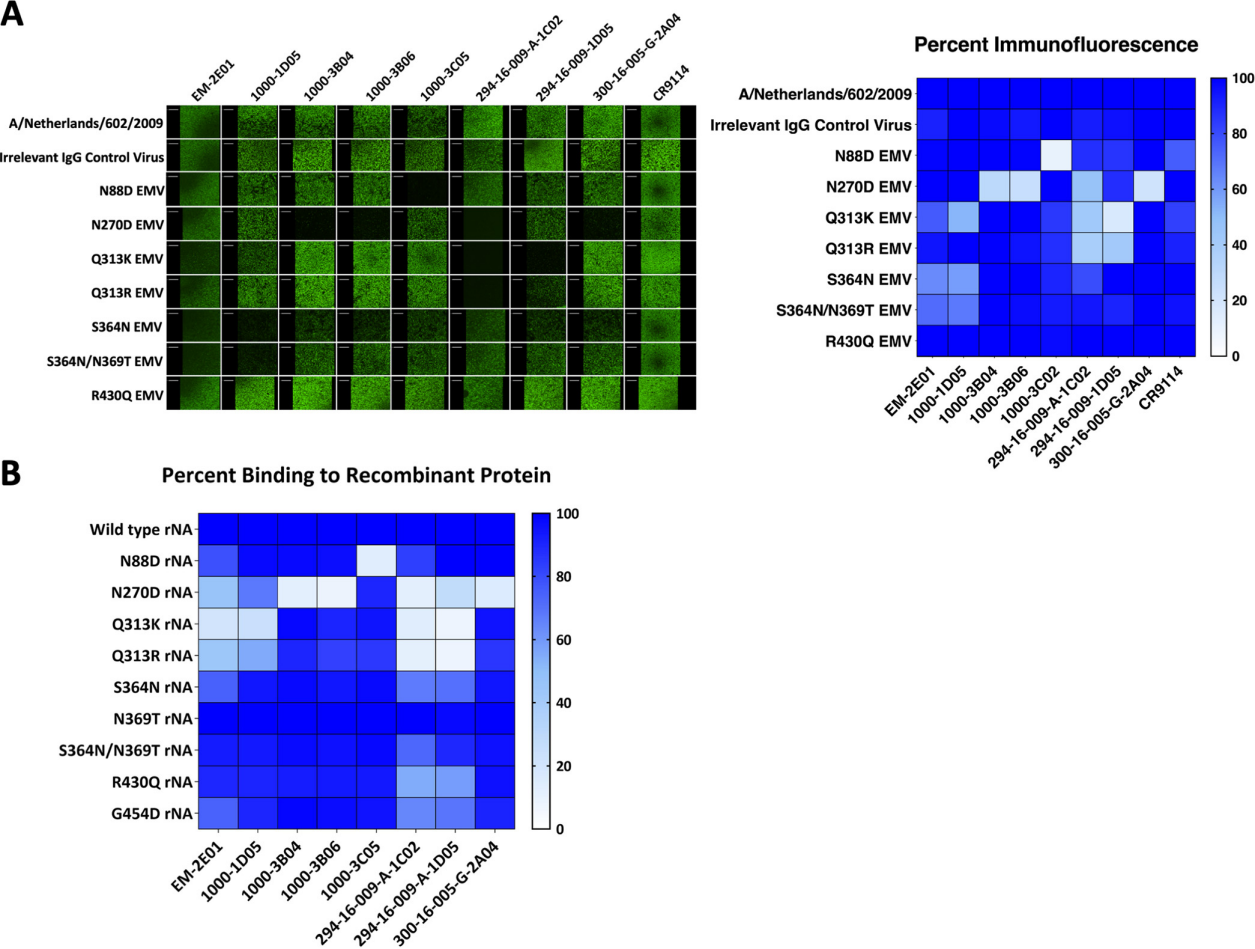
mAbs exhibit changes in binding activity toward EMVs. (A) Immunofluorescence assay comparing the binding of each mAb to the wild type and EMVs. On the left are representative images, and the right shows a heat map of percent fluorescence compared to the wild type. On the heat map, high binding is indicated by darker blue shading. Immunofluorescence assay images are representative of results from 2 independent experiments. (B) Percent binding of mAbs to rNA proteins determined using an ELISA. ELISAs were performed in triplicate, with average percent binding reported in the figure.

We next used the mAbs to evaluate the impact of each NA mutation on antibody binding, NAI, and neutralization activities. Using immunofluorescence assays, we identified that the N270D mutation impacted most of the antibodies in our panel, including those that produced other EMVs (Fig. 2A). The N88D mutation affected the binding of only the mAb that caused the mutation, 1000-3C05. Q313K impacted the binding of the mAb that caused that mutation (294-16-009-A-1C02) and mAb 294-16-009-A-1D05, while Q313R impacted the binding of only 294-16-009-A-1C02. S364N and the double mutation S364N/N369T affected the binding of EM-2E01 and 1000-1D05 (Fig. 2A). All mAbs retained binding to the R430Q EMV. Additionally, recombinant NA (rNA) proteins containing each EMV mutation were generated, and mAb binding was assessed using an enzyme-linked immunosorbent assay (ELISA). We observed similar changes in binding toward rNAs, with N270D impacting the binding of a majority of mAbs in the panel (Fig. 2B). N88D impacted the binding of only 1000-3C05. Interestingly, Q313K and Q313R rNAs exhibited altered binding with EM-2E01 and 1000-1D05, along with 294-16-009-A-1C02 and 294-16-009-A-1D05. Of note, reduced binding has also been reported for 1000-1D05 for an N309S mutant, which is a position close to Q313 (14). We observed a slight loss of binding of EM-2E01 to S364N rNA; however, the N369T and the double mutant S364N/N369T rNAs maintained mAb binding. Both R430Q and G454D had little impact on overall mAb binding, although reduced binding was observed for 294-16-009-A-1C02 and 294-16-009-A-1D05.

**FIG 3:**
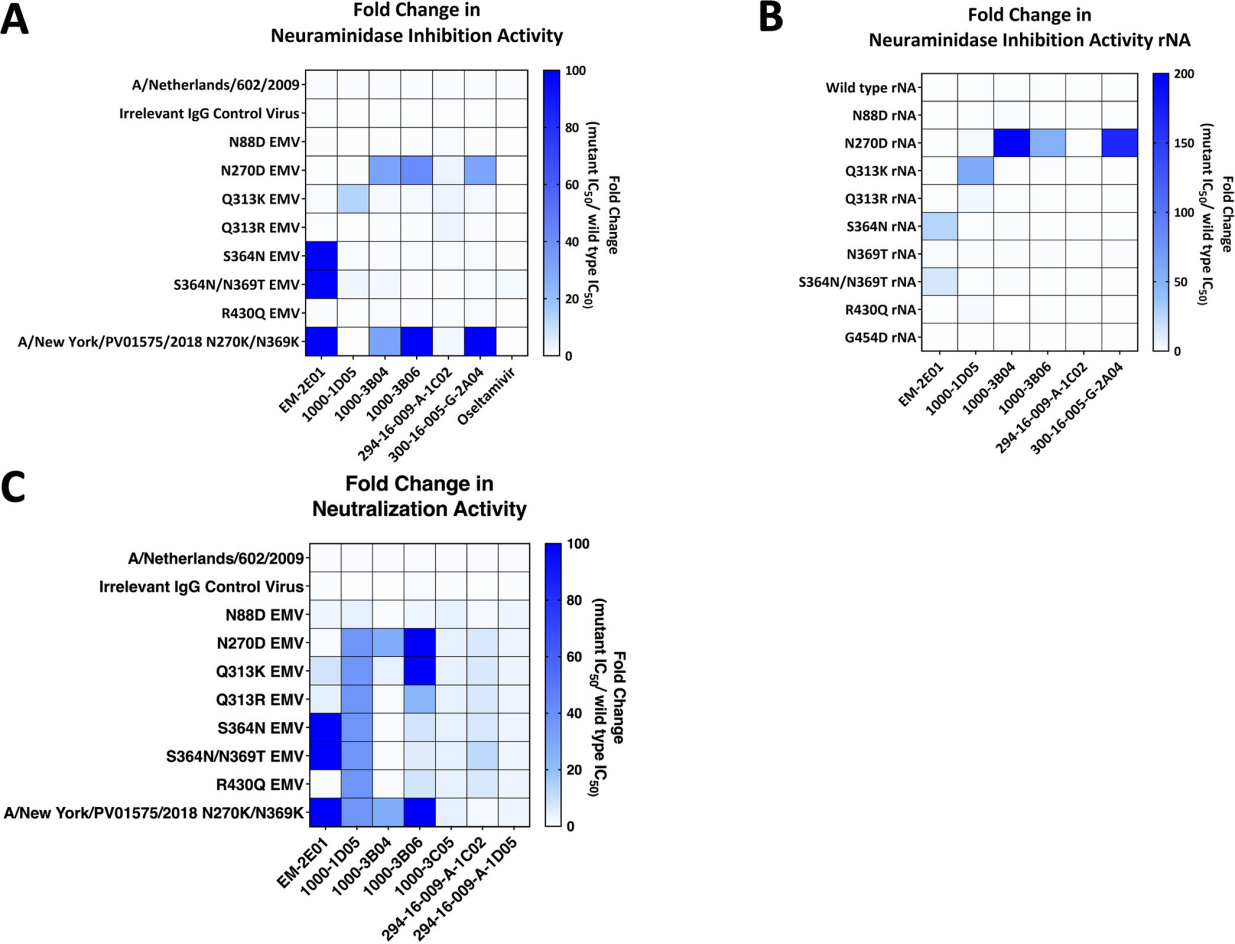
mAbs exhibit changes in NAI and neutralizing activities toward EMVs. (A and B) Heat maps of fold changes in NAI IC_50_s of each mAb against EMVs (A) or rNA proteins (B). (C) Fold changes in neutralization IC_50_s of each mAb against EMVs. Darker blue is a higher fold change in the IC_50_, which indicates stronger escape phenotypes. NAI and neutralization assays were conducted in duplicate.

To determine changes in NAI activity, we performed ELLAs with each EMV in the presence of each mAb. Additionally, we performed NAI assays using rNA proteins. While 1000-3C05 has no NAI activity, we still assessed if the mutation that it potentially caused, N88D, impacted any other antibodies in the panel. We found no significant changes in the NAI activity of any of the mAbs tested with N88D (Fig. 3A and B and Table 3). We found that the N270D EMV exhibited complete escape from 1000-3B04 and 294-16-009-A-1C02, along with resistance to 1000-3B06 (42-fold increase in the NAI IC_50_) and 300-16-005-G-2A04 (30-fold change in the NAI IC_50_) (Fig. 3A and Table 3). The N270D rNA exhibited a similar phenotype, with complete escape from 1000-3B04, 1000-3B06, and 294-16-009-A-1C02 along with resistance to 300-16-005-G-2A04 (168-fold change in the NAI IC_50_) (Fig. 3B). The Q313K EMV and rNA became resistant to the NAI activity of 1000-1D05 (13-fold and 57-fold increases in NAI IC_50_s, respectively) and completely escaped from 294-16-009-A-1C02. Both the Q313R EMV and rNA completely escaped 294-16-009-A-1C02 without causing resistance to other mAbs (Fig. 3A and B and Table 3). S364N and S364N/N369T EMVs completely escaped from EM-2E01 (Fig. 3A and Table 3). The S364N and S364N/N369T rNAs also became resistant to EM-2E01 (26-fold and 14-fold increases in NAI IC_50_s, respectively). However, the N369T rNA did not exhibit resistance to any mAbs in the panel. The mutation R430Q did not have a strong impact on any mAb NAI activity (Fig. 3A and B). We also identified a natural isolate, A/New York/PV01575/2018, which contained mutations at residues identified in EMVs (N270K and N369K) (Table 2). This virus completely escaped EM-2E01, 1000-3B04, 1000-3B06, and 300-16-005-G-2A04 (Fig. 3A). No EMVs showed increased resistance to the neuraminidase inhibitor oseltamivir (Fig. 3A and Table 3).

**TABLE 3:** NAI IC_50_ values for mAbs against all EMVs^*a*^

Virus/rNA	Inhibitory activity (μg/mL) (95% confidence interval)						
	EM-2E01	1000-1D05	1000-3B04	1000-3B06	294-16-009-A-1C02	300-16-005-G-2A04	Oseltamivir
Irrelevant IgG control virus B	0.012 (0.011–0.014)	0.076 (0.059–0.098)	0.54 (0.48–0.62)	0.15 (0.13–0.18)	5.65 (4.61–6.99)	0.059 (0.052–0.066)	0.087 (0.073–0.092)
N88D EMV	0.018 (0.015–0.021)	0.078 (0.60–0.10)	0.70 (0.56–0.86)	0.23 (0.20–0.26)	12.03 (7.76–22.18)	0.067 (0.057–0.079)	0.051 (0.046–0.057)
N270D EMV	0.014 (0.011–0.016)	0.50 (0.29–0.90)	>30*	11.25 (8.68–15.21)	>30*	2.97 (2.21–4.10)	0.088 (0.069–0.11)
Q313K EMV	0.032 (0.030–0.035)	8.50 (6.61–11.21)	1.66 (1.40–1.98)	0.45 (0.37–0.55)	>30*	0.16 (0.14–0.19)	0.15 (0.13–0.18)
Q313R EMV	0.015 (0.013–0.017)	0.11 (0.074–0.18)	0.77 (0.67–0.89)	0.19 (0.16–0.26)	>30*	0.066 (0.052–0.082)	0.070 (0.062–0.080)
S364N EMV	>30*	0.90 (0.62–1.31)	1.02 (0.78–1.34)	0.32 (0.23–0.44)	11.77 (9.34–15.40)	0.13 (0.097–0.16)	1.259 (0.87–1.86)
S364N/N369T EMV	>30*	1.61 (1.00–2.67)	2.04 (1.61–2.61)	0.29 (0.21–0.41)	8.05 (5.69–11.84)	0.13 (0.088–0.19)	2.62 (2.19–3.15)
R430Q EMV	0.017 (0.016–0.022)	0.14 (0.10–0.20)	1.05 (0.85–1.30)	0.24 (0.18–0.33)	8.56 (5.72–13.90)	0.11 (0.093–0.12)	0.056 (0.050–0.064)
A/New York/PV01575/2018 N270K/N369K	>30*	0.041 (0.029–0.056)	>30*	>30*	19.79 (14.02–30.00)	>30*	0.064 (0.056–0.073)
Wild-type rNA	0.020 (0.019–0.021)	0.12 (0.11–0.13)	0.013 (0.013–0.014)	0.19 (0.17–0.21)	>10	0.027 (0.025–0.028)	ND
N88D rNA	0.0092 (0.0075–0.011)	0.11 (0.092–0.12)	0.0057 (0.0034–0.0082)	0.21 (0.16–0.26)	>10	0.027 (0.024–0.030)	ND
N270D rNA	0.0081 (0.0069–0.0093)	0.42 (0.29–0.61)	9.20 (5.11–34.06)***	>10***	>10	3.66 (2.92–4.64)	ND
Q313K rNA	0.019 (0.017–0.021)	5.89 (4.74–7.46)**	0.0126 (0.0095–0.017)	0.17 (0.14–0.21)	>10	0.024 (0.022–0.025)	ND
Q313R rNA	0.015 (0.013–0.017)	0.56 (0.46–0.68)	0.013 (0.011–0.014)	0.17 (0.16–0.20)	>10	0.024 (0.023–0.025)	ND
S364N rNA	0.38 (0.23–0.61)	0.088(0.074–0.10)	0.0192 (0.013–0.028)	0.12 (0.064–0.22)	>10	0.015 (0.012–0.020)	ND
N369T rNA	0.044 (0.041–0.048)	0.19 (0.17–0.22)	0.0198 (0.017–0.023)	0.27 (0.22–0.32)	>10	0.033 (0.031–0.035)	ND
S364N/N369T rNA	0.18 (0.11–0.31)	0.11 (0.075–0.17)	0.019 (0.015–0.025)	0.074 (0.045–0.12)	>10	0.017 (0.015–0.019)	ND
R430Q rNA	0.011 (0.0012–0.10)	0.24 (0.13–0.44)	0.0006384 (2.83 × 10^−6^–0.14)	0.16 (0.041–0.69	>10	0.0167 (0.015–0.018)	ND
G454D rNA (corresponds to irrelevant IgG control virus A)	0.0066 (0.0051–0.0084	0.038 (0.030–0.049)	0.01237 (0.008875–0.01670	0.061 (0.025–0.13)	>10	0.020 (0.016–0.025)	ND

a IC_50_ values (with 95% confidence intervals) are listed in micrograms per milliliter. Thirty micrograms per milliliter was the highest mAb concentration assessed for EMVs, while 10 μg/mL was the highest mAb concentration tested for rNAs. Boldface type indicates significant differences in IC_50_s between irrelevant IgG control virus B and the EMV (*, *P* < 0.05; **, *P* < 0.01; ***, *P* < 0.001). ND, not determined.

We observed a more significant impact on mAb neutralization than on NAI activity. This may be caused by the mechanism of neutralization, which relies on strong NAI activity to prevent virus spread and plaque formation. Small changes in NAI activity could allow the formation of plaques, resulting in increased neutralizing IC_50_ values in a plaque reduction neutralization assay, where each plaque present is counted regardless of its size. The N88D EMV completely escaped mAbs 1000-3C05 and 294-16-009-A-1D05 (Fig. 3C and Table 4). The N270D EMV had complete escape from all mAbs in the panel aside from EM-2E01 (Fig. 3C and Table 4). Q313K led to escape from 1000-1D05, 1000-3B06, 1000-3C05, 294-16-009-A-1C02, and 294-16-009-A-1D05. However, Q313R led to escape from 1000-1D05, 1000-3C05, 294-16-009-A-1C02, and 294-16-009-A-1D05 but did not impact the neutralization activity of 1000-3B06. S364N and S364N/N369T EMVs completely escaped EM-2E01, 1000-1D05, 1000-3C05, 294-16-009-A-1C02, and 294-16-009-A-1D05 (Fig. 3C and Table 4). We found that the R430Q EMV escaped 4 mAbs, 1000-1D05, 1000-3C05, 294-16-009-A-1C02, and 294-16-009-A-1D05 (Fig. 3C and Table 4). A/New York/PV01575/2018 escaped all mAbs in the panel aside from 294-16-009-A-1C02. Importantly, irrelevant IgG control virus B had NAI and neutralization IC_50_ values similar to those of the wild-type virus, and a recombinant NA featuring the D454G mutation of irrelevant IgG control virus A also did not directly impact mAb activity (Tables 1, 3, and 4).

**TABLE 4:** Neutralization IC_50_ values for mAbs against all EMVs^*a*^

Virus	Neutralization activity (μg/mL) (95% confidence interval)						
	EM-2E01	1000-1D05	1000-3B04	1000-3B06	1000-3C05	294-16-009-A-1C02	294-16-009-A-1D05
Irrelevant IgG control virus B	0.027 (0.030–0.030)	0.34 (0.28–0.42)	0.85 (0.83–0.86)	0.20 (0.11–0.30)	14.98 (10.47–20.83)	5.59 (4.57–6.84)	22.34 (7.35–38.32)
N88D EMV	0.087 (0.067–0.15)	11.40 (7.89–16.27)	2.65 (1.27–5.69)	0.41 (0.26–0.63)	>100	25.56 (18.98–34.88)	>100
N270D EMV	0.046 (0.040–0.051)	>100*	>100*	>100*	>100	>100*	>100
Q313K EMV	0.28 (0.24–0.32)	>100*	15.21 (8.06–26.27)	>100*	>100	>100*	>100
Q313R EMV	0.16 (0.15–0.16)	>100*	4.32 (4.08–4.57)	3.48 (3.01–3.84)	>100	>100*	>100
S364N EMV	>100*	>100*	4.10 (4.00–4.20)	1.20 (0.73–2.63)	>100	>100*	>100
S364N/N369T EMV	>100*	>100*	4.50 (1.07–13.99)	0.81 (0.74–0.87)	>100	>100*	>100
R430Q EMV	0.032 (0.031–0.033)	>100*	4.20 (4.11–4.30)	1.15 (0.82–1.91)	>100	>100*	>100
A/New York/PV01575/2018 N270K/N369K	>100*	>100*	>100*	>100*	>100	24.40 (16.02–33.72)	>100

a Values were determined using PRNAs. IC_50_ values (with 95% confidence intervals) are listed in micrograms per milliliter. One hundred micrograms per milliliter was the highest mAb concentration tested. Boldface type indicates significant differences in IC_50_s between irrelevant IgG control virus B and the EMV (*, *P* < 0.05).

### Characterization of escape mutant viruses *in vivo*.

Evaluating the *in vivo* fitness of each EMV is important to determine if natural isolates that acquire these mutations will have a probability of showing increased or decreased fitness. However, since the EMVs created through passaging acquired mutations outside the NA, likely including cell culture-adaptive mutations, it is not possible with this set of viruses to determine which mutations directly impact fitness. Nevertheless, we still went ahead with these experiments because the outcome can at least indicate if any of the NA mutations severely attenuate the EMVs *in vivo*. We determined the mouse 50% lethal dose (mLD_50_) of each EMV to assess fitness changes *in vivo*. Since both Q313R EMV and S364N EMV had similar changes in binding, NAI, and neutralization activities to Q313K and S364N/N369T EMVs, only one was chosen for mLD_50_ experiments. Wild-type A/Netherlands/602/2009 (H1N1)pdm09 virus had an mLD_50_ of 1 PFU (Table 5). A majority of EMVs had mLD_50_ values similar to that of the wild-type virus, including the N88D, N270D, Q313K, and S364N/N369T EMVs (Table 5). The R430Q EMV had a moderate (45-fold) increase in the mLD_50_ (Table 5). However, we also noted that this EMV contained a unique HA mutation, K226M. As explained above, this experiment has significant caveats. However, it suggests that none of the mutations detected severely attenuated the EMVs. This is different than what has been observed with similar EMVs generated against stalk-binding antibodies where severe attenuation *in vivo* was detected (35).

**TABLE 5:** Lethal doses of EMVs in mice

Virus	mLD_50_ (PFU/mouse)
A/Netherlands/602/2009 (H1N1)pdm09	1
N88D EMV	6
N270D EMV	18
Q313K EMV	8
S364N/N369T EMV	2
R430Q EMV	316

## DISCUSSION

Our study has identified novel epitopes on the N1 targeted by human mAbs. Only 2 of the escape mutations detailed here have been previously reported, N270 and N369, indicating that these 2 residues are frequently targeted epitopes of human mAbs (14, 27, 30). Interestingly, a majority of the 2017–2018 isolates contained N270K and N369K mutations, which further emphasizes their importance for mAb binding and NAI activity. The remaining mutations, N88D, Q313K/R, and S364N, are part of newly identified mAb epitopes.

The mutation N88D, a critical residue for 1000-3C05, is located very close to the NA head-stalk interface. mAb 1000-3C05 does not exhibit NAI activity and is poorly neutralizing; however, previous reports have noted that it is protective *in vivo*, is cross-reactive with several human N1s (pre- and postpandemic), and can utilize Fc effector functions (14, 31). The position of N88 on the protein may explain why 1000-3C05 is NAI inactive as this mAb does not interfere with the enzymatic site of the NA.

Mutations at Q313 were necessary for the evasion of 294-16-009-A-1C02 during escape mutagenesis. Once the EMV was identified, we noted that this residue was also important for another mAb in our panel, namely, 1000-1D05. This residue is located on the side of the NA, outside any previously defined antigenic regions. We noticed that the Q313K mutation had a slightly stronger effect on mAb escape than Q313R. This may be best explained by amino acid biochemistry. Lysine has a lesser degree of freedom to form electrostatic interactions as compared to arginine and this 'rigidity' could lead to stronger interruption of antibody-antigen interactions.

We identified two separate EMVs containing the mutation S364N. When comparing escape phenotypes, both the S364N and S364N/N369T EMVs completely escaped the mAb EM-2E01, became resistant to the neutralization of 1000-1D05, and remained sensitive to the remaining mAbs in the panel. These data suggest that S364N is sufficient for escape from EM-2E01 and that N369T is not critical for mAb escape, which was confirmed with recombinant NA carrying either S364N or N369T. Furthermore, the S364N mutation alone introduces an N-linked glycosylation site, which is likely responsible for blocking mAb activity. This is interesting because many recent isolates contain mutations at N369, like the A/New York/PV01575/2018 isolate used in this study, but S364 is highly conserved.

To truly understand how these mutations impact NA antigenicity, it would be important for future studies to test how human sera inhibit the neuraminidase activity of our EMVs. Nevertheless, our findings can already aid in annual vaccine strain selection since they can help to identify amino acid changes in circulating strains that may lead to antigenic drift of the NA. In addition, our findings can also inform the design of NA-based vaccines (35, 36). Specifically, they indicate which amino acids are important for interactions with human antibodies. This information helps in the design of immunogens in which epitopes that include these amino acid positions are displayed correctly. Conversely, it can also help in the design of antigens in which the targeting of these amino acid positions is avoided since they are prone to change.

Anti-NA antibodies can inhibit NA through several mechanisms. They can bind directly to the active site, thereby blocking the access of the substrate to it. They can also bind further away and block interactions between the active site and substrate through steric hindrance. Finally, it could also be hypothesized that antibodies may bind far away from the enzymatic site, triggering allosteric changes that incapacitate the active site. As described previously by Chen et al. (14), EM-2E01 inhibited the NA in both an NAI assay using sialic acid attached to an N-linked glycan on large, bulky fetuin as the substrate as well as an NAI assay using a small-molecule substrate. This suggests that the mAb binds directly to the active site. The fact that EM-2E01 drove escape mutation S364N, which is close to the active side, supports this hypothesis. All other mAbs used here (with the exception of 300-16-005-G-2A04) exhibit activity only in NAI assays with fetuin but not in assays with a small-molecule substrate (14), suggesting binding outside the enzymatic site and inhibition via steric hindrance. Again, this is supported by the escape mutations that we found for these mAbs.

In addition to mutations in the NA gene, most EMVs also contained mutations R62K, D239G, and R240Q in their HA. Both D239G and R240Q have been previously reported to increase virus growth *in vitro* (37, 38). The mutation R62K has not yet been fully characterized; however, it has been observed in natural isolates and may be involved in HA stability (39). Both the N270D and Q313K EMVs shared the HA mutation K136N, which has not yet been described in the literature. The R430Q EMV acquired a unique HA mutation, K226M, which has also not been previously characterized. Based on our *in vivo* fitness data, it appears that this HA mutation could have a slight impact on viral fitness. Importantly, the R62K, K163N, D239G, and R240Q mutations were also found in one of the control viruses that was passaged without pressure from NA-specific mAbs, suggesting that these mutations represent cell culture-adaptive mutations. The second control virus contained an HA mutation, E391G, which was also present in the A/New York/PV01575/2018 isolate. The NP mutation S50N present in both the Q313K EMV and the R430Q EMV has been identified as a mutation that does not impact polymerase activity *in vitro*, leading us to conclude that this mutation does not have an impact on the viral fitness observed (40). The PA mutations identified in these viruses, V100L and V407I, have not yet been characterized in the literature. Additionally, the NP mutation E372D and the M1 mutation E204D, which were identified only in the N88D EMV, have not previously been reported. Based on the N88D EMV phenotype compared to the wild-type virus, we do not suspect that these mutations have a significant impact on viral fitness. However, since we did not test the mutations in isolation, it is not possible to state if or to what degree they contributed to the escape phenotype observed. The only statement that can be made about the escape mutants is that according to *in vivo* data, the mutations in the NA did not result in a strongly attenuated phenotype. This is in contrast to what we have observed with the same virus strain when we used a similar approach to map anti-HA stalk mAbs (34). Escape from antistalk mAbs caused a strong attenuation phenotype in many EMVs.

Combined with data from previous reports, we can conclude that human antibodies are targeting more than just the enzymatic site (14, 30–32). Figure 1 illustrates where the EMV mutations, along with others discussed here, are located on the NA. Aside from overlaps at positions 248, 249, 270, 273, 309, 369, 451, and 456, the epitopes for murine mAbs are unique compared to what has been observed for human mAbs. This highlights why it is important to evaluate antigenic sites using human monoclonal antibodies to increase our understanding of how the N1 is being targeted by our immune responses.

## MATERIALS AND METHODS

### Cells, virus, and antibodies.

MDCK cells (ATCC CCL-34) were obtained from the American Type Culture Collection (ATCC) and propagated using complete Dulbecco’s modified Eagle medium (cDMEM) (1× Dulbecco’s modified Eagle medium [Gibco], 10% heat-inactivated fetal bovine serum [Sigma-Aldrich], 1-U/mL penicillin–1-μg/mL streptomycin solution [Gibco], and 10 mM 2-[4-(2-hydroxyethyl)piperazin-1-yl]ethane-1-sulfonic acid (HEPES) [Gibco]).

A/Netherlands/602/2009 (H1N1)pdm09 was grown in our laboratory by injection of 10-day-old specific-pathogen-free (SPF) embryonated chicken eggs (Charles River Laboratories) and incubation at 37°C for 2 days. All mAbs were identified and isolated previously and provided by Patrick Wilson (14). They were expressed in our laboratory using the Expi293 transfection kit according to the manufacturer’s instructions (Thermo Fisher). mAbs were purified by gravity flow with protein G-Sepharose-packed columns and concentrated as described previously (41).

### Escape mutant generation.

Escape mutant viruses were generated using the H1N1 virus A/Netherlands/602/2009 (H1N1)pdm09 as the maternal strain. MDCK cells were plated at 6 × 10^5^ cells/mL in a 12-well, sterile cell culture plate and incubated overnight at 37°C with 5% CO_2_. The following day, virus was diluted to an MOI of 0.01 (1 × 10^3^ PFU) in 1× minimal essential medium (MEM) (10% 10× MEM [Gibco], 2 mM l-glutamine [Gibco], 0.1% sodium bicarbonate [Gibco], 10 mM HEPES, 1-U/mL penicillin–1-μg/mL streptomycin solution, and 0.2% bovine serum albumin [BSA]) supplemented with 1 μg/mL tosylsulfonyl phenylalanyl chloromethyl ketone (TPCK)-treated trypsin. Antibodies were then added to the virus at a concentration that was 0.25 times the neuraminidase inhibition (NAI) 50% inhibitory concentration (IC_50_). For the mAbs without NAI activity (1000-3C05 and 294-16-009-A-1D05), the plaque reduction neutralization assay (PRNA) IC_50_ was used instead. The virus-antibody mixture was incubated with shaking for 1 h at room temperature (RT). MDCK cells were washed with 1× phosphate-buffered saline (PBS; Gibco), and the mixture was then added to the cells. They were then incubated at 37°C with 5% CO_2_ for 2 days. The supernatant was collected and stored at −80°C until further use. For subsequent passages, MDCK cells were plated as described above, infected with a 1:10 dilution of the previous passage in 1× MEM with TPCK-treated trypsin (1 μg/mL), and incubated for 40 min at 37°C with 5% CO_2_. In the meantime, mAb was diluted in 1× MEM with TPCK-treated trypsin to a concentration that was doubled from the previous passage (passage 1 was 0.25× IC_50_, passage 2 was 0.5×, and so on). After 40 min, diluted mAb was added to the virus-infected cells and left for 2 days at 37°C with 5% CO_2_. The cell culture supernatant was screened for escape mutant viruses by plaque assays with 128× IC_50_ of mAb present in the agarose overlay. Individual plaques (3 to 6 per EMV) were chosen and propagated in SPF eggs for 2 days at 37°C as described above.

### RNA isolation and deep sequencing.

RNA was isolated from egg allantoic fluid using the E.Z.N.A. viral RNA extraction kit (Omega Bio-Tek) according to the manufacturer’s instructions and then underwent next-generation sequencing. Sequences were assembled using a pipeline designed at the Icahn School of Medicine at Mount Sinai as described previously (42). To identify point mutations, full-length coding sequences were compared to the sequenced wild-type A/Netherlands/602/2009 (H1N1)pdm09 strain used for escape mutagenesis. All reported mutations were numbered from the methionine of each protein.

### Plaque assay.

Plaque assays were performed using a standard protocol. MDCK cells were seeded 24 h previously at 8 × 10^5^ cells/mL in a sterile, 12-well plate and incubated overnight at 37°C with 5% CO_2_. Next, virus samples were serially diluted in 1× MEM from 10^−1^ to 10^−6^. MDCK cells were washed with 1× PBS and then infected with 200 μL of each virus dilution. Virus was incubated for 40 min at 37°C with 5% CO_2_, with rocking every 10 min. Afterward, virus was aspirated and immediately replaced with 1 mL of an agarose overlay containing 2× MEM, 0.1% diethylaminoethyl (DEAE)-dextran, 1 μg/mL TPCK-treated trypsin, and 0.64% Oxoid agarose. Plates were incubated for 2 days at 37°C with 5% CO_2_. Cells were then fixed using a 3.7% solution of paraformaldehyde (PFA) and incubated at 4°C overnight. For plaque visualization, the overlay was removed, and cells were stained with a solution containing 20% methanol and 0.5% crystal violet.

### ELISA.

Recombinant NA proteins of A/California/7/2009 (H1N1) with mutations were produced using the well-established baculovirus expression system, which has been described in detail previously (43). Immulon 4HBX 96-well microtiter plates (Thermo Fisher Scientific) were coated overnight at 4°C with recombinant proteins (100 ng/well) in PBS (pH 7.4). The well contents were discarded and blocked with 200 μL of 3% nonfat milk (American Bio) in PBS containing 0.1% Tween 20 (PBST) for 1 h at RT. After blocking, 50 μL of monoclonal antibodies diluted (starting at an Ig concentration of 10 μg/mL and serially diluted 3-fold) with 1% nonfat milk in PBST was added to each well for reaction at RT for 2 h. After washing with PBST three times, 50 μL of horseradish peroxidase (HRP)-labeled goat anti-human IgG(H+L) cross-adsorbed secondary antibody (Thermo Fisher Scientific) diluted 2,500-fold with 1% nonfat milk in PBST was added to each well, and the mixture was incubated at RT for 1 h. After washing with PBST three times, 100 μL of SigmaFast *o*-phenylenediamine dihydrochloride (OPD) substrate solution (Sigma-Aldrich) was added to each well for the reaction at RT for 10 min. The reaction was stopped by the addition of 50 μL of 3 M hydrochloric acid (HCl). The optical density at 490 nm was measured using a Synergy 4 plate reader (BioTek). The area under the curve (AUC) was calculated in GraphPad Prism 9. Percent binding was determined by comparing the wild-type rNA AUC to mutant rNA AUC values.

### Immunofluorescence.

MDCK cells were plated at 3 × 10^5^ cells/mL in a sterile, 96-well plate and incubated overnight at 37°C with 5% CO_2_. The following day, cells were checked for >99% confluence and washed with 1× PBS. Virus was diluted to an MOI of 5 in 1× MEM and added to each well (100 μL/well). Plates were incubated overnight at 37°C with 5% CO_2_. The following day, cells were fixed using 200 μL of 3.7% PFA and incubated overnight at 4°C. Next, the PFA was removed, and cells were blocked with 1× PBS containing 3% nonfat milk (American Bio) for 1 h at room temperature. The blocking solution was then removed and replaced with 1% nonfat milk. Primary mAbs were diluted to 300 μg in 1× PBS and added to the 1% milk at a 1:10 dilution, for a final concentration of 30 μg per well. Primary antibodies were incubated with shaking for 1 h at room temperature. Plates were then washed 3 times with 1× PBS. The secondary antibody Alexa Fluor 488 goat anti-human IgG(H+L) (Invitrogen) was diluted to 1:500 in 1% milk and added to the plates, and the mixture was incubated for 1 h at room temperature in the dark with shaking. The plates were then washed 3 times with 1× PBS. To prevent cells from drying out, 50 μL of 1× PBS was added to each well. Plates were visualized using the Celigo S adherent cell cytometer (Nexcelom Bioscience) with the 2-channel target 1+2 (merge) setting. The exposure time, gain, and focus (set using image-based autofocus with the 488-nm signal as the target) for the 96-well plate were automatically determined by the machine. Fluorescence was calculated using the default analysis settings, and percent fluorescence was determined based on the wild-type signal. We performed 2 independent assays; however, representative images from only 1 assay are shown here. The mAb CR9114 was included as a positive control to illustrate that all viruses had similar infectivities. This mAb is a pan-influenza A virus (IAV) HA stalk mAb (44).

### Enzyme-linked lectin assay.

Flat-bottom Immulon 4HBX microtiter plates (Thermo Scientific) were coated with 25 μg/mL of fetuin (Sigma) diluted in 1× PBS, at 100 μL per well and incubated overnight at 4°C. The next day, viruses were serially diluted (3-fold) in sample diluent buffer (1× PBS with 0.5 mM MgCl_2_, 0.9 mM CaCl_2_, 1% BSA, and 0.5% Tween 20) in a sterile 96-well plate. Once diluted, an additional 1:1 ratio of the sample diluent was added to the plate. This mixture was incubated for 1 h at room temperature with shaking. After 1 h, the fetuin-coated plates were washed 3 times with PBST using the AquaMax 3000 automated plate washer. Diluted virus was immediately added to the plates, and the plates were then incubated at 37°C with 5% CO_2_ for 18 h (overnight). The following day, plates were washed 6 times with PBST. Peanut agglutinin (PNA; Sigma) was diluted to 5 μg/mL in conjugate diluent buffer (1× PBS with 0.5 mM MgCl_2_, 0.9 mM CaCl_2_, and 1% BSA) and added to the washed plates. This mixture was incubated for 2 h in the dark at room temperature. The PNA was then removed, and plates were washed 3 times with PBST. SigmaFast OPD (Sigma) was diluted in water. OPD was added at 100 μL per well, and the mixture was incubated for 3 min at room temperature. The reaction was stopped by adding 50 μL of 3 M hydrochloric acid, and the absorbance (at 490 nm) was then immediately determined using a Synergy H1 hybrid multimode microplate reader (BioTek). Prism 7.0 was used to determine the effective concentration of each virus that would yield detectable NA activity. Each enzyme-linked lectin assay (ELLA) was done in triplicate.

### Neuraminidase inhibition assay.

To determine the NAI activity of each mAb, flat-bottom Immulon 4HBX microtiter plates (Thermo Scientific) were coated with 25 μg/mL fetuin (Sigma) diluted in 1× PBS and incubated overnight at 4°C. The following day, antibodies were diluted in sample diluent buffer and then serially diluted 1:3 in a sterile 96-well plate. Virus or rNA was diluted to 2 times the effective concentration determined by an ELLA and added to mAbs at a 1:1 ratio. This mixture was incubated for 1 h at room temperature with shaking. The fetuin-coated plates were washed 3 times with PBST as described above. Virus-mAb dilutions were transferred to the fetuin-coated plates and incubated at 37°C, with 5% CO_2_, for 18 h (overnight). The following day, we performed the ELLA procedure as described above. The IC_50_ and 95% confidence intervals were determined using a nonlinear regression [log(inhibitor) versus response-variable slope (4 parameters)] in Prism 7.0. Each NAI assay was performed in duplicate. Significance between IC_50_ values for each EMV and the irrelevant IgG control virus was calculated using 2-way analysis of variance (ANOVA). The fold change in the mAb NAI IC_50_ was calculated by dividing the EMV IC_50_ by the IC_50_ of irrelevant IgG control virus B. Fold changes in NAI IC_50_s for rNAs were calculated by dividing the rNA IC_50_ by the wild-type rNA IC_50_.

### Plaque reduction neutralization assay.

MDCK cells were plated at 8 × 10^5^ cells/mL in 12-well plates. The following day, mAbs were diluted to 100 μg/mL in 300 μL 1× MEM and serially diluted 1:5 in 1× MEM to a final concentration of 0.032 μg/mL. Each virus was then diluted in 1× MEM to 1 × 10^3^ PFU, and 50 μL was added to each antibody dilution. This virus-mAb mixture was incubated for 1 h at room temperature with shaking. Afterward, MDCK cells were washed with 1× PBS and then immediately infected with 200 μL per well of the virus-mAb mixture. The plates were incubated for 40 min at 37°C with 5% CO_2_, rocking every 10 min. In the meantime, the overlay was prepared. Antibodies were diluted to 100 μg/mL in 625 μL of 2× MEM and then serially diluted as described above. Next, a solution containing 1× DEAE-dextran and 1 μg/mL of TPCK-treated trypsin in sterile water for injection (Gibco) was added at 180 μL to each antibody dilution. When the infection finished, 360 μL of 2% Oxoid agarose was added to the overlay mixture in small batches to prevent solidification before being transferred to cells. The inoculum was removed and immediately replaced with the overlay so that the mAb dilution in the overlay was the same as the concentration in the inoculum. The plates were then incubated at 37°C, with 5% CO_2_, for 2 days. Cells were fixed and stained as described above. When determining plaque numbers per well, all plaques present were counted regardless of their size. The IC_50_ and 95% confidence intervals were determined using a nonlinear regression [log(inhibitor) versus response-variable slope (4 parameters)] in Prism 7.0. Each PRNA was done in duplicate. Significance between neutralizing IC_50_ values for each EMV and the irrelevant IgG control virus was calculated using 2-way ANOVA. The fold change in the mAb PRNA IC_50_ was calculated by dividing the EMV IC_50_ by the IC_50_ of irrelevant IgG control virus B.

### Mouse lethal dose.

The 50% mouse lethal dose (mLD_50_) for each EMV was determined using female BALB/c mice (at 6 to 8 weeks of age; Jackson Laboratory) in accordance with protocols approved by the Institutional Animal Care and Use Committee at the Icahn School of Medicine at Mount Sinai. Each virus was diluted from 10^5^ to 10^1^ in 1× PBS, and 3 mice per dilution were infected (50 μL per mouse). Weight loss and survival were monitored daily for 14 days postinfection. Mice that lost more than 25% of their initial body weight were euthanized.
